# Endocrine Abnormalities in Children With Traumatic Brain Injury at a Tertiary Care Center

**DOI:** 10.7759/cureus.34131

**Published:** 2023-01-24

**Authors:** Aaida Rao, Altaf Ali Laghari, Iman Bari, Muhammad Usman Khalid, Salman Kirmani, Muhammad Ehsan Bari

**Affiliations:** 1 Neurosurgery, Aga Khan University Hospital, Karachi, PAK; 2 Surgery, University of New South Wales, Sydney, AUS; 3 Pediatrics and Child Health, Aga Khan University Hospital, Karachi, PAK

**Keywords:** traumatic brain injury, pakistan, pediatric head injury, endocrine pathology, hypopituitarism

## Abstract

Objective

Accidental traumatic brain injury (TBI) can lead to severe complications such as endocrine abnormalities and long-term morbidities and can negatively impact patient lives. These conditions are also associated with a high cost of treatment over a lifetime, a significant concern in low-to-middle-income countries (LMICs). In Pakistan, the prevalence of children with endocrine abnormalities secondary to TBI remains largely unexplored. We conducted a retrospective cross-sectional study to estimate the burden of endocrine abnormalities due to TBI among children in our population.

Methods

Twenty patients previously admitted with head injury between September and October 2019 were retrospectively reviewed with tests for baseline serum sodium, plasma osmolality, cortisol, adrenocorticotropin (ACTH), free thyroxine (fT4), growth hormone (GH), insulin growth factor-1 (IGF-1), follicle-stimulating hormone (FSH), luteinizing hormone (LH), thyroid-stimulating hormone (TSH), prolactin, estradiol, and testosterone. Data were collated from the electronic Health and Information Management System (HIMS) and analyzed using SPSS v25. Chi-square and t-tests were used to identify associations between variable groups. Outcomes of interest included correlations between hormonal levels and demographic factors, interventions and hormonal levels, and complication rates and hormonal levels.

Results

Our study reports three (15% of the total cohort) patients with pituitary hormone deficits (two with low IGF-1 and one with low TSH). High serum IGF-1 and ACTH levels were also observed in three (15%) children. High IGF-1 was associated with female gender (p=0.007), mechanical ventilation (p=0.038), and falls (p=0.028). IGF-1 (p=0.035) and GH (p=0.049) levels were associated with improvement in Extended Glasgow Outcome Scale (GOS-E) score. Testosterone was positively correlated with a high percentile for height (p=0.005) and GOS-E scores on follow-up (p=0.030). High testosterone levels (592.12 ± 102.28 ng/dl) were associated with good functional outcomes in post-pubescent patients (p<0.05). Serum fT4 was linked with a high GOS-E score at discharge in prepubescent patients (p=0.034). Neurosurgical decompression was the only risk factor for hormone deficiency, comprising 67% of the group with hormone deficiencies (p=0.028). The learning difficulties were observed exclusively in children with hormonal deficiencies (7 patients, p=0.000).

Conclusion

Hormonal dysfunction due to TBI in children can lead to poor outcomes. High serum IGF-1, testosterone, and free T4 levels were associated with improved functional outcomes in children with TBI. Limited follow-up and resources in LMICs are significant barriers to addressing the morbidity associated with these conditions and need to be addressed at a health policy level.

## Introduction

Traumatic brain injury (TBI) occurs when an external force on the cranium leads to transient or permanent neurological dysfunction [1]. Amongst all traumatic injuries, brain injury carries the highest morbidity and mortality [1]. With around 1% of all children aged 14 and younger affected, the CDC in the United States estimates that TBI is the leading cause of lifelong disability among children and young adults [2]. Many mild and moderate complications of TBI can be diagnosed early and optimally treated [3]. However, some severe sequelae, such as hypopituitarism, reported in more than 25% of patients with TBI in literature, can have lifelong implications and a significant impact on the quality of life patients lead [4-6]. Several mechanisms are responsible for developing hypopituitarism following TBI, with hypophysial vessel injury being one of the most cited causes in literature. The external force can disrupt the long and vulnerable hypophyseal portal vessels supplying the anterior pituitary, impairing the hypothalamus-pituitary axis and causing dysregulation of pituitary hormone secretion [5]. Of note, Benvenga S initially described post-TBI hypopituitarism in a patient with a basilar skull fracture in 1918 [7]. In this context, several authors have reported impaired growth, precocious/delayed puberty, and other neuroendocrine abnormalities among pediatric patients following head injury [8,9]. However, the nature of these complications is vague on examination and evident very late in the course of the disease as patients attain puberty or adulthood [8]. This poses an additional burden to the caregivers and can adversely impact the patient's quality of life. Early screening for the detection of hypopituitarism in children with TBI and subsequent hormonal replacement therapy can obviate this problem. There is a wide variation in the prevalence of hypopituitarism in children with accidental TBI in literature (7%-60%), generally due to different hormonal testing techniques, study design, and patient demographics [10-13]. However, the burden of post-TBI hypopituitarism among South Asian children is still yet to be determined. In this study, we seek to assess the burden of post-traumatic hypopituitarism and its association with functional outcomes in pediatric patients admitted to one of Pakistan's highest-volume tertiary care centers.

## Materials and methods

Data source and study population

Patients with head injuries admitted to the ED of the Aga Khan University Hospital, Karachi, between September and October 2019 were identified. Our cohort study included all pediatric patients (age <18 years) admitted to the hospital with evidence of intracranial injury on a CT scan at the time of injury. Patients were excluded if they had a previous history of hormonal abnormalities, menstrual irregularities, substance abuse, chemo/radiotherapy, chronic kidney disease, or were taking steroids, oral contraceptive pills, or insulin. After obtaining institutional ethical approval (ERC:201913614072), the Health Information Management Systems (HIMS) department provided access to the patient's medical records. Data fields collected and entered into the database included demographic factors such as age, sex, height, and weight. In addition, clinical factors such as mode of injury, GCS, CT scan findings, hospital length of stay (LOS), ICU admission, neurosurgical decompression, and mechanical ventilation were also recorded. The severity of TBI was categorized into mild (GCS = 13-15), moderate (GCS = 9-12), and severe (GCS ≤ 8). Detailed written informed consent from the parents and written assent from the patients were acquired. Twenty patients were selected for final inclusion through a consecutive sampling technique.

Auxology 

The patient's pubertal status was determined by asking about menarche or secondary sexual characteristics (appropriate to gender at birth). The patient's height was measured to the nearest 1 cm, whereas the patient's weight was measured to the nearest 0.1 kg. The recorded height and weight were converted to percentiles using standardized growth charts. The patient's BMI was also calculated and classified according to Cole TJ et al. reference data for BMI [13]. A neurosurgeon assessed each patient's functional status using the Glasgow Outcome Scale Extended (GOS-E) at discharge and follow-up. Based on the GOSE score, functional outcome was categorized into two groups: scores 1-6 as 'Poor Recovery' and scores 7-8 as 'Good Recovery' [14]. 

Hormonal testing

Serum tests were performed on follow-up visits at six months, as part of the study protocol, for sodium, plasma osmolality, cortisol, adrenocorticotropic hormone (ACTH), free thyroxine (fT4), thyroid-stimulating hormone (TSH), growth hormone (GH), insulin-like growth factor-1 (IGF-1), follicle-stimulating hormone (FSH), luteinizing hormone (LH), prolactin, estradiol, and testosterone. The patients were in eight-hour fasting from 12 am to 8 am prior to testing. Additionally, urinary sodium, urine osmolality, and urine-specific gravity were recorded. The hospital's regional reference ranges according to age and sex were used to determine the abnormal hormone levels. 

Statistical analysis

Summary statistics, including frequency and percentage for categorical variables and the mean and SD for continuous variables, were reported. Fisher's exact test and independent samples t-test were used to compare categorical and continuous variables. Pearson correlation was used to assess associations between continuous variables. Statistical significance was defined as priori as a p < 0.05. All data analyses were performed using the SPSS, v.25 (IBM Corp, Armonk, NY, USA).

## Results

Patient characteristics at the time of injury 

A total of 20 participants consisting of three (15%) females and 17 (85%) males were included in the study. The mean age at injury was 12 ± 5 years, and the mean age at testing was 15 ± 5 years. Eight (40%) patients were prepubescent, and 12 (60%) were postpubescent. Road traffic accidents (RTA) (16, 80%) and falls (4, 20%) were the causes of head injury in our cohort. Prepubescent patients comprised the majority (12, 75%) of patients admitted with TBI due to falls. Severe TBI was diagnosed in 11 (55%) children, compared to patients with moderate (7, 35%) and mild (2, 10%) severity. Twelve (60%) patients were admitted to the ICU, nine (45%) patients needed mechanical ventilation, and only four (20%) children underwent neurosurgery. CT findings in patients included intracranial hemorrhage (11, 55%), vault fracture (14, 70%), basilar skull fracture (8, 40%), and contusion at the site of trauma (12, 60%). Only five (25%) patients presented signs of high intracranial pressure (ICP) on ED admissions, such as midline shift, cerebral edema, basal cistern, and ventricular effacement. The average time since trauma was 25 months ± 7 months (IQR: 9-36 months). GCS was found to have a negative correlation with the length of stay, with a Pearson's correlation coefficient (PCC) of -0.848, p<0.001. 

Patients' characteristics at the time of follow-up testing 

Serum Hormone Levels

**Figure 1:**
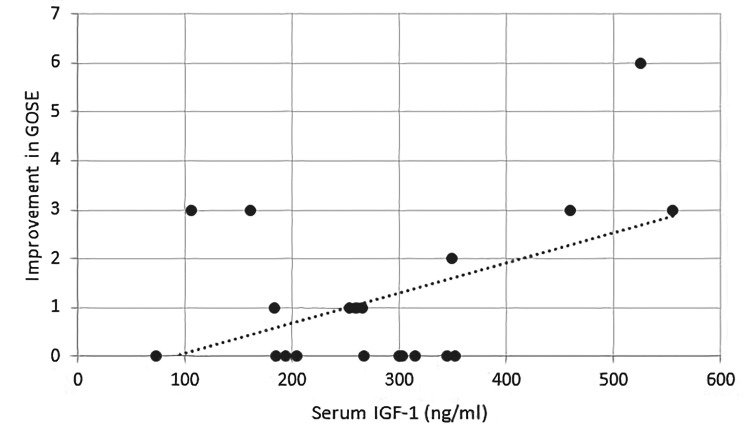
Positive correlation between IGF-1 and improvement in GOSE scores. IGF-1: Insulin growth factor-1; GOSE: Glasgow Outcome Scale Extended.

**Figure 2:**
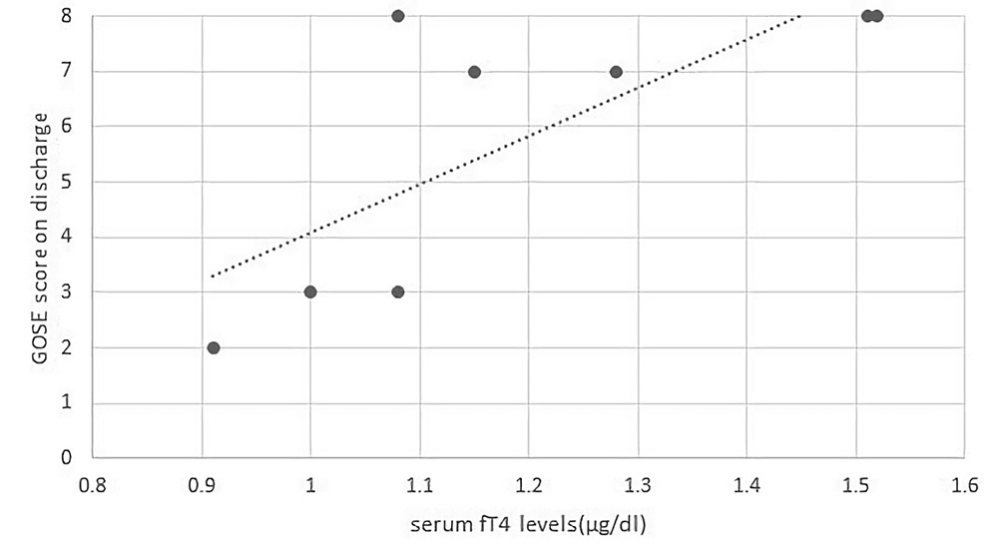
Positive correlation between fT4 and GOSE scores on discharge in pre-pubescent patients. GOSE: Glasgow Outcome Scale Extended; fT4: Free thyroxine.

Overall, no significant difference in the serum levels of hormones was observed between patients with good and poor outcomes (Table 1). However, among postpubescent patients, testosterone had a positive correlation with GOSE score on follow-up (PCC=0.682, p=0.030) and percentile for height (0.804, p=0.005). The mean serum testosterone levels were also significantly high in postpubescent patients with good outcomes (592.12 ± 102.28 ng/dl) as compared to those with poor outcomes (421.77 ± 58.2 ng/dl) (p<0.05). GH had a positive correlation with improvement in GOSE score with a PCC of 0.445 (p=0.049). IGF-1 also showed a positive correlation with improvement in GOSE score from discharge to last follow-up (with a PCC of 0.474, p=0.035) (Figure 1). IGF-1 was positively correlated with GH (with a PCC of 0.614, p=0.004), LH (with a PCC of 0.461, p=0.041), and testosterone (0.577, p=0.015). In prepubescent children, serum IGF-1 had a positive correlation with a PCC of 0.745 (p=0.034) with a percentile for BMI. Serum fT4 had a positive correlation with GOSE scores on discharge in prepubescent patients (PCC = 0.763, p=0.028) (Figure 2). 

**Table 1:** Mean (+/- SD) serum hormone levels stratified by outcome status. ACTH: Adrenocorticotropin; fT4: Free thyroxine; GH: Growth hormone; IGF-1: Insulin growth factor-1 (IGF-1); FSH: Follicle-stimulating hormone; LH: Luteinizing hormone; TSH: Thyroid-stimulating hormone.

Hormone	Good Outcome	Poor Outcome	P-value
Prolactin (ng/ml)	8.37(+/-4.51)	8.34(+/-5.28)	0.992
Estradiol (pg/ml)	67.95(+/-17.37)	0	no p-value obtained
Testosterone (ng/dl)	362.56(+/-298.19)	321.20(+/-186.41)	0.736
LH (mIU/ml)	2.47(+/-1.82)	3.17(+/-2.71)	0.614
FSH (mIU/ml)	5.53(+/-3.97)	4.42(+/-3.58)	0.578
GH (ng/ml)	0.66(+/-1.01)	0.23(+/-0.36)	0.173
IGF-1 (ng/ml)	299.03(+/-133.59)	233.54(+/-99.44)	0.274
Cortisol (µg/dl)	10.32(+/-3.84)	9.34(+/-2.99)	0.572
ACTH (pg/ml)	28.05(+/-13.06)	33.64(+/-19.64)	0.577
FT4 (µg/dl)	1.17(+/-0.21)	1.10(+/-0.11)	0.369
TSH (mIU/ml)	2.08(+/-1.12)	2.17(+/-1.03)	0.871

Endocrine Abnormalities 

Three (15%) of the patients presented pituitary hormone deficiencies. Other endocrine abnormalities included high IGF-1 (3, 15%) and high ACTH (3, 15%). Significantly fewer patients with endocrine abnormalities had basilar skull fracture (12.5% vs. 58.3%, p=0.040) or vault skull fracture (37.5% vs. 91.6%, p=0.010) on CT scans as compared to those without endocrine abnormalities. Most of the patients with endocrine abnormalities received intensive care at the time of injury (87.5% vs. 41.7%, p=0.040) and had learning difficulties (62.5% vs. 16.7%, p=0.034) as compared to those without endocrine abnormalities. Most patients with normal ACTH levels had a healthy BMI compared to those with slightly high baseline ACTH levels on testing (76.4% vs. 0%, p=0.017). 

Hormonal deficiencies with associated patient characteristics

Low hormonal levels were only identified in four (20%) postpubescent boys. Low IGF-1 levels were found in two (10%) patients, while isolated low TSH level was found in one patient. We observed one case of isolated low fT4 levels with normal TSH levels, but the abnormal levels were not due to altered pituitary function. One 17-year-old boy that previously presented to the ED with severe TBI (GCS 3) with brainstem contusion and subarachnoid hemorrhage (SAH) had hemiparesis at the time of discharge. Fourteen months later, he had partial resolution of the motor deficit but developed learning difficulty along with obesity, stunted height (3rd percentile), and high ACTH and low IGF-1 at the testing time. Another patient with low serum IGF-1 levels was a 19-year-old boy who presented with severe TBI (GCS 5 due to severe contusion) and frontotemporal fracture. He also presented with a learning difficulty and a 30th-percentile of height at the time of testing. A 15-year-old boy with frontal contusion and SAH due to severe TBI (GCS 4) presented with isolated low TSH levels with behavioral problems at the time of testing. Significantly more patients with hormonal deficiencies underwent neurosurgical decompression (67% vs. 11.7%, p=0.028) and developed a learning and behavioral problems (100% vs. 0%, p= 0.000) compared to those without hormonal deficiencies.

High IGF-1 Levels

High IGF-1 levels were identified in three (15%) patients, including two prepubescent children. All three patients with high IGF-1 levels on testing previously had major radiographic abnormalities on CT scans and had presented with hemiparesis, dysphagia, and dysphonia at the time of injury. Two out of three of these patients had developed an overweight BMI status at the time of testing. The majority of the patients with high IGF-1 levels were females (67% vs. 5.8%, p=0.007), suffered from TBI due to falls (67% vs. 11.8%, p= 0.028), and required mechanical ventilation (100% vs. 35%, p=0.038) at the time of injury compared to those with normal and low serum IGF-1 levels. Most of these patients later developed learning disabilities (67% vs. 5.8%, p=0.013) and fine motor deficits (75% vs. 25%, p=0.000) at the time of testing compared to those with normal and low serum levels of IGF-1. 

Functional outcomes

The average GOSE score was 6 ± 2 on discharge and 7 ± 1 on follow-up. The majority of these patients (16, 80%) had a GOSE score of 8 on follow-up. Persistent complications due to TBI were reported in eight (40%) patients at the time of testing. TBI complications included fine motor deficits in 10 (50%) of the patients, followed by neurocognitive disorders (including behavioral problems, learning deficits, and dysgraphia) in seven (35%) patients.

## Discussion

In our experience, 15% of pediatric patients with TBI developed isolated long-term pituitary hormone deficiency, including two with low IGF-1 levels and short stature and one with low TSH levels. We compared our findings with studies that used hormonal baseline tests as frontline testing in their protocols; Salomón-Estébanez MA et al. found four out of 36 patients with low serum IGF-1 levels but without any growth impairment, while Moon RJ et al. found only one case of delayed puberty. However, the delayed puberty was later discovered due to the carbamazepine the patient was taking and was thus not counted as hypopituitarism [15,16]. In comparison to these studies, Bellone S et al. reported the highest number of patients with pituitary hormone deficits despite having a majority of patients with a mild head injury. Although four patients in the study were diagnosed with hypopituitarism, only one case of low IGF-1 level was identified [17]. GHD remains one of the most common endocrine deficiencies with TBI, as reported in previous literature and our study [11]. Bellone S et al. found that 29% of the children showed poor growth velocity (<25% percentile) six months after injury, and 65% of them continued to exhibit poor growth a year later [17]. Unfortunately, we were unable to measure the height velocity in our study, as it requires more than one patient visit. We did, however, observe that between the two patients in our sample with low IGF-1 levels, one presented with a height of the 3rd percentile a year after injury, whereas the other patient attained a height of the 30th percentile three years after injury. We speculate that IGF-1 may eventually rise to normal levels in the subsequent years in patients with TBI-induced GHD. However, a longitudinal study will be required to confirm this speculation. Nonetheless, proper growth during childhood and adolescence is crucial in attaining a normal permanent height in adulthood. Some studies have shown persistently poor growth in children with TBI during their growth spurt [17].

A unique finding in our study was the surprisingly high IGF-1 level in three children with severe TBI. These patients exhibited hemiparesis, dysphagia, and dysphonia at the time of injury that fully resolved on follow-up except for some fine motor deficits and learning difficulty. To our knowledge, few studies have reported incidences of high IGF-1 levels in children with TBI as yet [16-18]. Interestingly, serum GH and IGF-1 levels in our patients strongly correlated with the improvement of GOSE scores from discharge to follow-up. This may be due to GH's capacity to increase the number of neurons that result in the brain growth-promoting in vivo actions of the hormone [18]. Moreover, numerous clinical trials have supported using GH replacement therapy for TBI rehabilitation in adults. However, instances of hyperglycemia and fluid retention in GH replacement therapies and the epileptic potential of IGF-1therapy have rendered GH infusion unsuitable for children [19-22]. Apart from IGF-1's effect on functionality, prepubescent children with high IGF-1 also had a high percentile for weight and were significantly overweight compared to those with low or normal IGF-1 levels. Hatton J et al. reported that TBI patients undergoing IGF-1 therapy started to gain weight and developed a positive nitrogen balance, a finding corroborated by Bettendorf M [23,24]. In TBI-induced GHD, severe stress due to head injury could result in a state of GH resistance that leads to decreased production of IGF-1, which mediates the anabolic effects of the GH. Therefore, patients with GHD undergo a hypercatabolic state for up to several months after trauma resulting in poor growth and, thus, poor functionality [21-25]. Other pituitary hormones have also shown an association with functionality in our study. Serum testosterone had a positive correlation with high IGF-1 levels and a high percentile for height. Testosterone also was associated with a high GOSE score on follow-up and good functional outcomes in postpubescent patients. Hypothyroidism due to TBI is known to lead to developmental delay and neuropsychiatric effects in children [6,12,23]. In prepubescent patients, a high GOSE score on discharge was positively correlated with high serum fT4 on follow-up in our study. Thyroxine (fT4) may also be a marker for good functionality in children with a head injury like testosterone. However, this claim would have more weight if the serum fT4 levels were also measured at the time of discharge. Furthermore, one of the patients had persistent low TSH levels 18 months after injury, despite hypothyroidism being mostly an acute complication of TBI. 

Some studies identified a link between abnormal CT findings and endocrine abnormalities, while others found no association between CT findings and endocrine abnormalities [26-28]. Our study reported that patients with basilar or vault skull fractures had fewer cases of endocrine abnormality than those without basilar or vault skull fractures. We also did not find any significant association between endocrine abnormalities and the severity of TBI. At the same time, some studies reported a higher frequency of hypopituitarism in patients with severe TBI compared to those with moderate TBI [29]. We observed that more patients with endocrine abnormalities received intensive care compared to those with normal hormone levels, as reported by previous studies [4,30]. In addition to the above risk factors, more patients who later developed endocrine abnormality underwent neurosurgery decompression compared to patients without hormonal deficits. This claim is supported by another study that reports the need for surgical intervention had the greatest odds ratio as a potential risk factor for pituitary dysfunction [31]. LMICs are resource-limited by definition, and endocrine abnormalities typically have long-term ramifications in terms of treatment and quality of life [28]. A major reason for a cross-sectional study design and overall lack of data has been the high lost-to-follow-up rates in our region [32]. Financial elements in nutrition and growth (such as the cost of providing adequate nutrition for overpopulated and impoverished families) can be a big factor in the poor long-term outcomes observed. They must be mitigated through a stronger support system at both the governmental and healthcare service system levels. Telehealth is an emerging field that may help increase follow-up due to the increasing availability of smartphones and connectivity, even in rural areas.

Limitations

One of the limitations of this study was the small sample size which might have led to the underreporting of patients with hypopituitarism. For instance, Bellone S et al. conducted baseline tests on 70 patients and detected four cases of hypopituitarism, whereas Moon RJ et al., on the other hand, conducted baseline tests on 20 patients and found no patients with hypopituitarism [16,17]. Secondly, our study had significant selection bias as most of our study sample consisted of patients with severe and moderate injuries. Significantly few parents of children with mild head injury were willing to enroll their child in our study as they believed the injury was not serious enough to warrant further testing. Thirdly, we could not confidently confirm patients with abnormal baseline hormonal tests with TBI-induced hypopituitarism due to the lack of dynamic hormonal testing. Although most studies use dynamic testing to diagnose hypopituitarism, our study did not include these tests due to hypoglycemia and high false positive rates in patients [33-36]. We could, however, use dynamic testing as confirmatory tests on patients with abnormal baseline hormonal values and intend to follow this approach in our subsequent studies on post-TBI hypopituitarism. Lastly, this cross-sectional study design was unable to record transient endocrinopathies and the mean time interval of testing in a few of our patients was very long (36 months). 

## Conclusions

Our findings suggest that high levels of serum IGF-1, testosterone, and fT4 may serve as possible markers for good functional outcomes in children with TBI. By conducting a larger prospective study with a hormonal investigation at regular intervals along with GOSE scoring, we may be able to identify the correct time to administer hormone replacement therapy in these children to improve their functional outcomes.
